# Transversus abdominis plane block with general anesthesia blunts the perioperative stress response in patients undergoing radical gastrectomy

**DOI:** 10.1186/s12871-019-0861-0

**Published:** 2019-11-07

**Authors:** Ruizhu Liu, Haiyan Qin, Meng Wang, Kai Li, Guoqing Zhao

**Affiliations:** 1 Department of Anesthesiology, China-Japan Union Hospital of Jilin University, No. 126 Xiantai Road, Changchun, 130000 Jilin Province China; 2 Department of Plastic Surgery, China-Japan Union Hospital of Jilin University, Changchun, 130000 Jilin Province China; 3 Department of Cardiology, No. 965 Hospital of PLA, Jilin, 132000 Jilin Province China

**Keywords:** Gastric cancer, Nerve block, Analgesia, Stress

## Abstract

**Background:**

Surgical stress induces the release of neuroendocrine mediators and cytokines during perioperative period, which may have adverse effects on cancer patients. While the surgical stress responsse can be affected by anesthetic technique. Therefore, we designed this study to assess whether subcostal transversus abdominis plane (TAP) block can affect perioperative neuroendocrine stress response, postoperative analgesia and postoperative recovery in patients undergoing radical gastrectomy under general anesthesia.

**Methods:**

Sixty-five patients were recruited. Patients randomly received general anesthesia (control group), or general anesthesia combined with TAP block (40 mL of 0.375% ropivacaine) (TAP group). The primary outcome was neuroendocrine levels including norepinephrine (NE), epinephrine (E), cortisol (Cor), glucose (Glu), interleukin (IL)-6 and IL-10 during 48 h after surgery. Secondary outcomes included pain score, hemodynamic variables and recovery characteristics.

**Results:**

Data from 61 of 65 patients were analyzed. The levels of NE, E, Cor, and Glu were blunt by TAP block during perioperative period. The levels of IL-6 and IL-10 were significantly lower in TAP group than in control group. TAP block efficiently relieved postoperative acute pain up to 12 h postoperatively with more stable perioperative hemodynamics compared with control group.

**Conclusions:**

Subcostal TAP block blunts perioperative stress response and provides efficient analgesia, with good hemodynamic stability and minimal adverse effects.

## Background

Gastric cancer is one of the most commonly diagnosed cancers around the world. Surgical resection is currently the mainstay of curative treatment for this cancer, which is recommended to maximize the probability of complete tumor clearance, reduce loco-regional recurrence, and improve long-term survival [1]. Transabdominal radical gastrectomy is an effective therapeutic strategy for malignant gastric disease. However, open gastrectomy causes severe pain in the incision of abdominal wall. Surgical trauma accompanied with considerable surgical pain is a key variable affecting the surgical stress response and prognosis [2]. The responses of patients towards surgical pain may result in unstable hemodynamics, and this excessive stress action is adverse for postoperative rehabilitation and long-term prognosis for patients with malignant tumor [3]. Thus, effective modulation of stress response may reduce the incidence of complications [4, 5].

Transversus abdominis plane (TAP) block, as an emerging regional anesthetic technique, can provide efficient analgesia by blocking the regulation of sensory nerve at the anterior abdominal wall [6], which can reduce opioid consumption, and lessen opioid-related side effects [7–9]. TAP block is given before the occurrence of noxious stimulation, which could relieve the postoperative pain by alleviating the pain of peripheral and central sensitization [10]. Recently, analgesic effect of TAP block has been widely studied, but the study associated with its efficiency on stress response that compared with general anesthesia is limited.

Subcostal, mid-axillary and lumbar triangle of Petit approaches are widely used in TAP block [11]. The bi-subcostal approach is associated with wide block area of spread (T7-L1) [12, 13], and could provide a satisfactory analgesic plane for transabdominal gastrectomy. The current study was aimed to investigate the effects of subcostal TAP block on stress response and analgesic effect in patients undergoing open radial gastrectomy under general anesthesia.

## Methods

### Patients and grouping

Our study was approved by the Ethics Committee of China-Japan Union Hospital of Jilin University (2016ks008) and was in accordance with good clinical practice and the guiding principles of the Helsinki Declaration. The written informed consents were obtained from all patients. The trial was registered prior to patient enrollment at clinicaltrials.gov (NCT03035916, principal investigator: Guoqing Zhao, Ruizhu Liu, Kai Li, date of registration: 01/27/2017).

Patients meeting the following inclusion criteria were enrolled: patients who were scheduled for open radical gastrectomy; patients who had no contraindication to local anesthetic, and patients whose American Society of Anesthesiologists’ (ASA) physical status I, II or III. Emergency patients and patients with preoperative infection, or patients with a history of immune disease, endocrine system disease, blood transfusion, or chemotherapy were excluded from this study.

Patients were randomly divided into two groups by a computerized random-number generator with 1:1 ratio for TAP group and control group. The pre-anesthetic interview was carried out by a single investigator to assess the eligibility of participants and to record the baseline data. Another anesthesiologist (not involved in study) who took the corresponding opaque envelope in the operating room performed the block and anesthesia induction, so he was unblinded to group allocation. A resident anesthetist who was blinded to randomization was responsible for collection of intraoperative data and blood. Postoperative data and blood were collected by another investigator who visited the participants at 1, 6, 12, 24, 48 h after surgery. All patients underwent surgery from the same operative team. The patients allocated to respective groups were administered as follows: TAP group: general anesthesia and ultrasonography-guided TAP block with 40 ml of 0.375% ropivacaine; Control group: standard general anesthesia (no sham intervention for TAP block).

### Operation process

No preoperative sedatives or analgesics were administered before the operation. Routine monitoring including electrocardiogram, invasive arterial blood pressure, pulse oximetry, and end-tidal carbon dioxide was established before anesthetic induction. All patients underwent standard general anesthesia induced by midazolam 0.02 mg/kg (Enhua Pharmaceutical Co., Ltd., Jiangsu, China), propofol 2.0–2.5 mg/kg (Fresenius Kabi Deutschland, Germany), sufentanil 0.3 μg/kg (Yichang Humanwell Pharmaceutical Co., Ltd., Yichang, China), and cis-Atracurium 0.15 mg/kg (Hengrui Pharmaceutical Co., Ltd., Jiangsu, China). In order to maintain general anesthesia, intravenous and inhaled volatile anaesthetics of nitrous oxide, sevoflurane (Maruishi Pharmaceutical Co., Ltd., Japan), sufentanil, and cis-Atracurium were used. During the operation, the MAC value was maintained within 1.2–1.5% by regulating the inhalation concentration of nitrous oxide and sevoflurane. Tidal volume and rate were adjusted to maintain an end-tidal PCO2 of 30–40 mmHg. When hemodynamic values increased more than 15% above pre-induction baseline values, an appropriate dose (0–10 μg) of sufentanil was used to intervene. Heart rate (HR) < 40 beats/min (bradycardia) was intervened with 0.5 mg atropine, and mean arterial blood pressure (MAP) < 60 mmHg (hypotension) was treated with 5 mg ephedrine. The body temperature was maintained between 36 °C and 37 °C during the operation.

TAP block was administered after anesthesia induction. In the TAP group, TAP block was performed bilaterally under ultrasound (SonoSite Portable M-Turbo, Sonosite Inc., Bothwell, UT, USA) guidance at 30 min before the surgical incision. The probe was placed in the midline of abdomen below the xiphoid and moved right laterally along the subcostal margin to the anterior axillary line. After the probe identified the plane between the rectus and transverse abdominal muscles, a 100-mm, 20-G Stimuplex block needle was guided within the plane to the point just inferior to the right costal margin such that the tip laid between the obliquus internus abdominis and transversus abdominis within the neurovascular fascial plane. After aspiration, to exclude vascular puncture, 1 mL test dose was injected to determine the flow resistance and confirm needle tip placement within the fascial plane. Then, 10 mL 0.375% ropivacaine was multi-point injected in this plane laterally along the subcostal margin. The probe was moved back to the anterior axillary line, and another 10 mL 0.375% ropivacaine was injected through the needle inferior to the costal margin within the TAP using an identical technique, making this two injected drug a continuous plane under the costal margin, from the medial margin of the rectus abdominis muscle to anterior axillary line. The left side of TAP block was conducted with the identical technique.

After extubation, patients were transferred to the post-anesthesia care unit (PACU), and all of them received morphine patient-controlled analgesia (PCA) with a standard dosing regimen: 2 mL/h continuous infusion (a bolus of 1 mL with a lock-time of 15 min) by using the electronics pump (Shanghai Bochuang, China).

### Postoperative detection

The primary outcome was neuroendocrine mediators and cytokines at different time points of 48 h after operation. Venous blood samples were collected before anesthesia (baseline), immediately after surgery, and at 6, 24, and 48 h after surgery, and stored in pre-chilled tubes on ice and then centrifuged within 90 min. The separated plasma was stored at − 80 °C. Glucose (Glu) level was measured immediately after venous blood was collected using glucometer (ACCU- CHEK Active, Inc.). Plasma levels of norepinephrine (NE), epinephrine (E), C-reactive protein (CRP), cortisol (Cor), interleukin (IL)-6, and IL-10 were measured by micro enzyme-linked immunosorbent assays kits (R&D system, Inc.,USA).

The second outcomes included pain scores, hemodynamic variables and recovery characteristics during the first 2 postoperative days. Pain scores included VAS scores at rest and during movement (cough or rotating the body) in the first 2 postoperative days. The MAP and HR were continuously measured and recorded before anesthesia, during induction, tracheal intubation, incision, immediately after tracheal extubation, as well as at 6, 24, and 48 h postoperatively. The other recorded observations included additional sufentanil consumption, postoperative opioid requirement, and recovery profiles (duration of PACU, side effects such as nausea, sedation, and vomiting, time of first flatus, and hospital stays). Sedation was assessed by Ramsay score (2–4: satisfactory sedation, > 4: excessive sedation). Additionally, discontinuing PACU was determined by a standard score test (= 10: PACU should be discontinued). The study processes are shown in Additional file 1: Figure S1.

The minimum detectable difference of plasma cortisol concentration was estimated immediately after surgery between two groups. The mean and SD values of plasma cortisol were as follows: MTAP = 410 nmol/L, MControl = 521 nmol/L, STAP = 142 nmol/L, and SControl = 16.4 nmol/L. Based on sample size analysis performed using PASS 11.0 (NCSS Statistical Software, Kaysville, UT), with α = 0.05 and β = 0.2, a minimum sample of 25 cases in each group was required, respectively.

### Statistical analysis

Statistical analyses were performed using SPSS 18.0 (Chicago, IL, USA). The normal distribution of data was evaluated using Shapiro-Wilk test. Data were expressed as mean (± SD) or percentages of the total number of patients (%). Comparisons of NE, E, Cor, Glu, cytokines, hemodynamic data, and VAS scores between groups were performed by repeated-measures analysis of variance. The differences for opioid consumption, time to first flatus, and time in PACU were analyzed using Student’s t test followed by 2-tailed Dunnett test. Categorical data were analyzed by using χ^2^ or Fisher exact test. *P* value < 0.05 was considered significant.

## Results

Total 65 patients were enrolled from February 2017 to August 2017. According to the exclusion criteria, 4 patients were excluded, and data from 61 patients (30 in TAP group, 31 in control group) were finally analyzed. Baseline characteristics showed no significant difference between two groups (Table 1).

**Table 1:** Demographic and perioperative characteristics by study group

Parameters	Control group (*n* = 31)	TAP group (*n* = 30)	*P*-value
Age (yr)	60.20 ± 8.93	58.95 ± 8.24	0.31
Gender (M/F)	23/8	21/9	0.75
Weight (kg)	65.90 ± 8.63	67.33 ± 11.62	0.42
Height (cm)	165.40 ± 12.10	168.90 ± 12.37	0.49
ASA physical status (I/II/III)	4/20/7	5/19/6	0.56
Duration of anesthesia (min)	235.25 ± 42.14	238.50 ± 43.66	0.79
Duration of surgery (min)	207.00 ± 34.01	195.83 ± 34.76	0.18

Values are mean ± SD or number

*ASA* American Society of Anesthesiologists

**Fig. 1:**
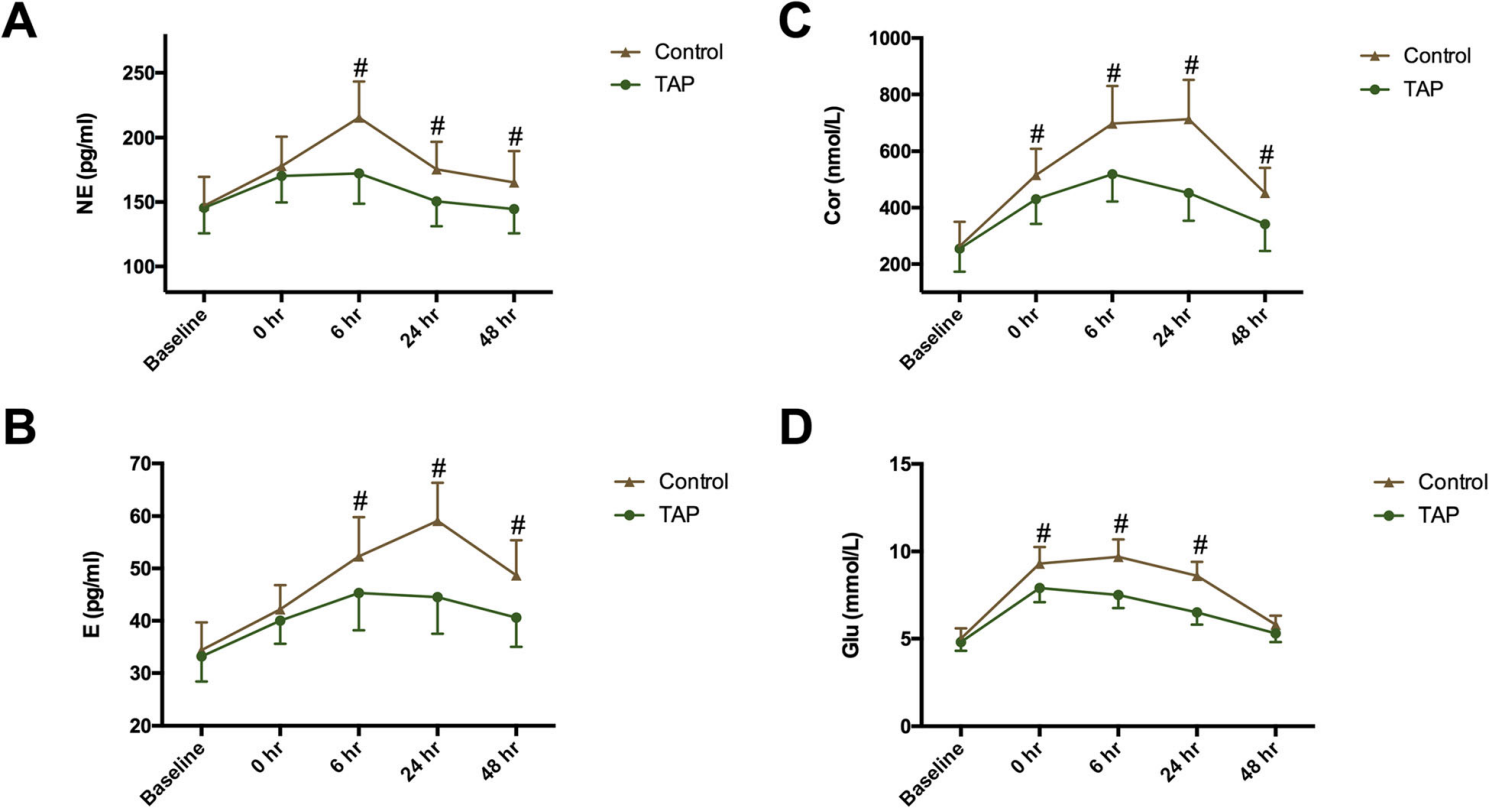
Plasma concentration of norepinephrine (NE) (**a**), epinephrine (E) (**b**), cortisol (Cor) (**c**), and glucose (Glu) (**d**). TAP: transversus abdominis plane. Time interval is defined as time between baseline and postoperative time. Data are expressed as mean ± SD. #: *P* < 0.05 compared with TAP group; *: *P* < 0.05 compared with baseline

There were no significant differences for NE, E, and Cor values on baseline. Compared with control group, the NE and E values were significantly lower in the TAP group at 6 h (P_NE_ = 0.001, P_E_ = 0.010), 24 h (P_NE_ = 0.022, P_E_ < 0.001) and 48 h postoperatively (P_NE_ = 0.001, P_E_ < 0.001) (Fig. 1a, b). The Cor levels were significantly higher in the control group than in TAP at all time points postoperatively (Fig. 1c). Additionally, the levels of NE, E, and Cor were more stable in TAP group compared with that in control group. Moreover, in TAP group, levels of the three hormones returned to baseline values by 24 h, whereas in the control group these parameters were significantly higher than the baseline values (Fig. 1). Glu levels did not differ between the two groups at baseline, but their levels were higher in the control group than in TAP group immediately after surgery, and at 6 h and 24 h postoperatively (*P* < 0.001) (Fig. 1d).

**Fig. 2:**
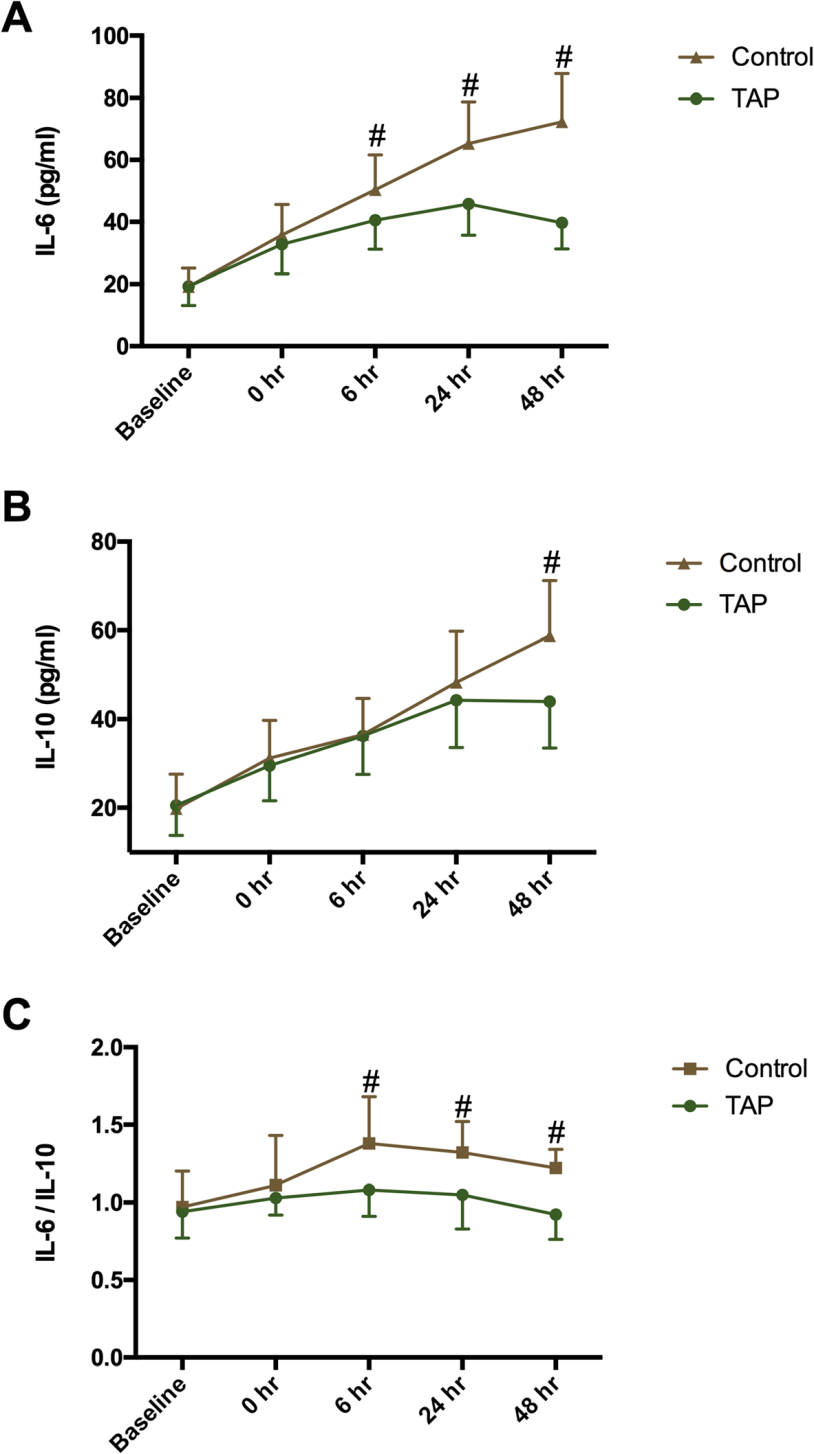
Serum concentration of interleukin (IL)-6 (**a**), IL-10 (**b**), and ratio of IL-6/IL-10 (**c**). TAP: transversus abdominis plane. Time interval is defined as time between baseline and postoperative time. Data are expressed as mean ± SD. #: P < 0.05 compared with TAP group; *: *P* < 0.05 compared with baseline

Plasma levels of IL-6 and IL-10 were not different at baseline. In the control group, IL-6 and IL-10 continued to increase even at 48 h postoperatively, whereas in TAP groups they peaked at 24 h postoperatively and then decreased (Fig. 2a, b). The IL-6 level was significantly lower in TAP group compared with in control group at 6 h (*P* = 0.0040), 24 h (*P* < 0.0001), and 48 h postoperatively (*P* < 0.001) (Fig. 2a). The IL-10 level was higher in the control group than in TAP group at 48 h postoperatively (*P* < 0.001) (Fig. 2b). Ratio of IL-6/IL-10 markedly greater in control group than in TAP group at postoperative 6 h, 24 h, and 48 h (all P < 0.001) (Fig. 2c).

**Fig. 3:**
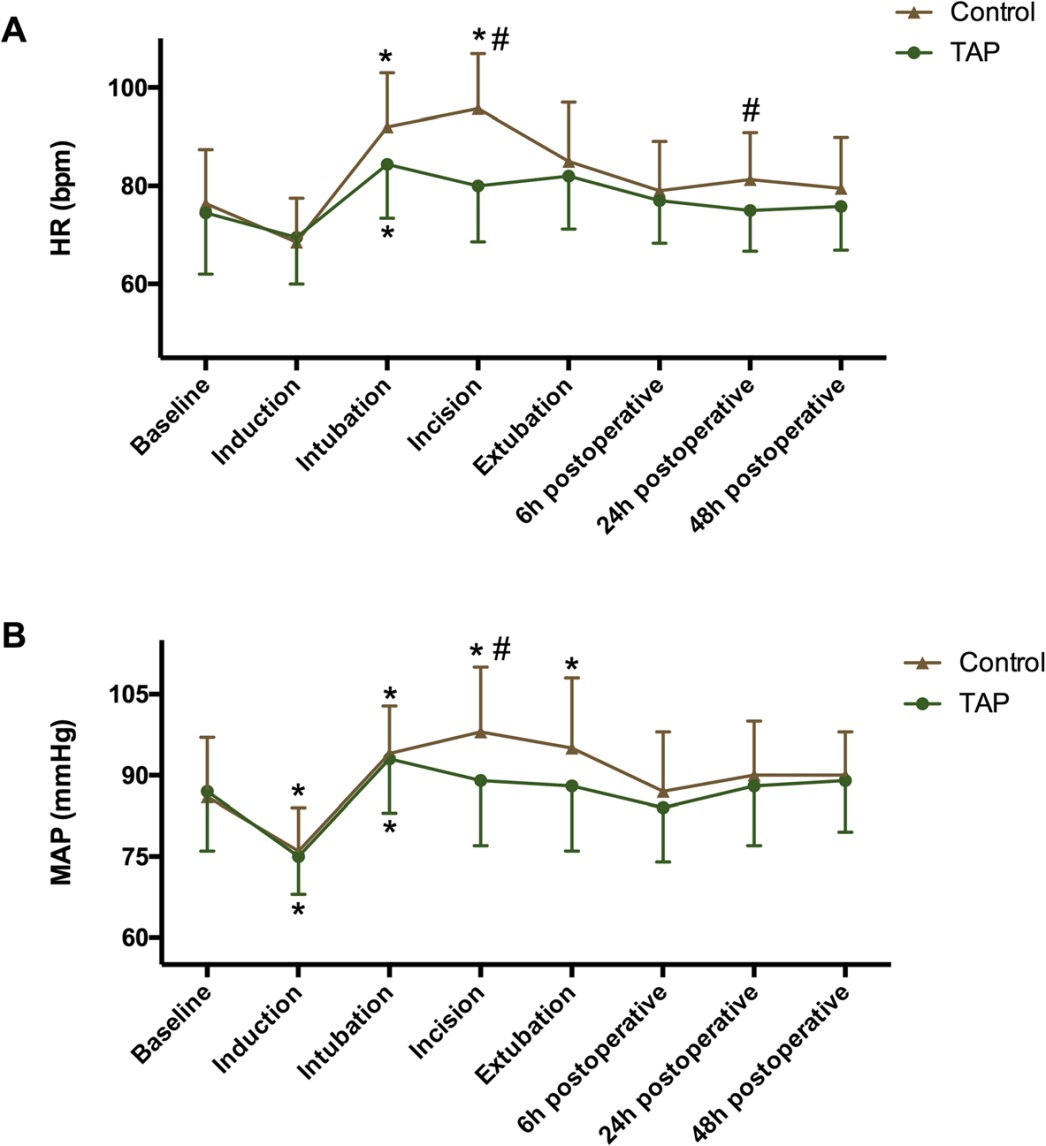
Hemodynamic changes during operation and 2 days postoperatively. **a** heart rate (HR); **b** mean arterial blood pressure (MAP). TAP: transversus abdominis plane. Data are expressed as mean ± SD. #: *P* < 0.05 compared with TAP group; *P < 0.05 compared with baseline

Pain scores for different time points are presented in Table 2. TAP group showed lower VAS scores at postoperative 1 h, 6 h, and 12 h whether the patients at rest or on movement. Whereas, no significant difference of VAS scores was found between TAP and control groups at postoperative 24 h and 48 h at rest. Hemodynamic data are shown in Fig. 3. In the control group, HR and MAP were higher at the incision time point relative to baseline and the TAP group (P_HR_ = 0.003, P_HR_ = 0.001; P_MAP_ = 0.002, P_MAP_ = 0.001). In TAP group, HR and MAP showed no significant difference relative to baseline except for induction and intubation time point.

**Table 2:** Visual Analog Scale Pain scores by study group

	TAP group *n* = 30	Control group *N* = 31	*P*-value
At rest			
1 h	2.2 ± 0.9	2.9 ± 0.9	0.017
6 h	2.2 ± 1.0	2.9 ± 1.0	0.045
12 h	2.1 ± 0.9	2.7 ± 1.2	0.008
24 h	2.2 ± 1.0	2.7 ± 1.0	0.175
48 h	1.0 ± 0.8	1.2 ± 0.8	0.417
On movement			
1 h	2.7 ± 1.2	4.3 ± 0.8	< 0.001
6 h	2.9 ± 0.7	4.2 ± 0.7	< 0.001
12 h	3.3 ± 0.5	4.3 ± 1.0	0.001
24 h	4.1 ± 0.6	4.6 ± 0.8	0.016
48 h	4.2 ± 0.8	4.5 ± 0.9	0.008

Data are presented as mean ± SD

Consumption of intraoperative sufentanil and postoperative morphine in TAP was significantly less compared with that in the control group (all *P* < 0.001) (Table 3). In control group 16 patients experienced sedation while in TAP group eight cases experienced. Additionally, patients in control group showed longer PACU stays, and hospital stays compared with TAP group. For time to first flatus, nausea, and vomit, no significant difference was identified between the two groups (Table 3).

**Table 3:** Doses of sufentanil, ephedrine, and nicardipine and recovery time

	Group TAP *n* = 30	Group Control *N* = 31	P-value
Opium consumption			
Intraoperative superaddition of sufentanil (μg)	4.3 ± 1.5	10.2 ± 2.8	< 0.001
Postoperative morphine consumption (mg)	46.6 ± 8.4	66.8 ± 10.9	< 0.001
Patients requiring ephedrine, n (%)	2, (6.7%)	1, (3.2%)	0.976
Time to first flatus (h)	77.6 ± 10.4	81.6 ± 10.4	0.131
PACU stay, (min)	44.2 ± 7.9	54.5 ± 8.2	< 0.001
Incidence of opium-related side effects, n(%)			
Sedation	8, (26.7%)	16, (51.6%)	0.046
Nausea	5, (16.7%)	6, (19.4%)	0.785
Vomiting	0, (0%)	1, (3.2%)	0.987
Long hospitalization, n(%)	5, (16.7%)	13, (41.9%)	0.031

Data are presented as mean ± SD or number;

Criteria for out of PACU: Aldrete scores ≥9;

Long hospitalization is defined as postoperative time ≥ 7 days

## Discussion

In this prospective, randomized trial, we found that the levels of NE, E, Cor, and Glu were blunt by TAP block during the perioperative period. Additionally, the levels of IL-6 and IL-10 were lower in TAP group significantly. Moreover, TAP block could efficiently relieve postoperative acute pain up to 12 h postoperatively with more stable perioperative hemodynamics compared with control group. Thus, TAP block can be used as an adjunct to general anesthesia to suppress stress responses and control pain in open radical gastrectomy surgery.

Perioperative stress response could be accurately indicated by changes of neuroendocrine mediators and cytokines via direct activation of the somatic and sympathetic nervous systems. It is well known that epidural block could block the sympathetic response and efficiently block the plasma levels of cortisol and relevant cytokines in patients [14–16]. TAP block is another method of pre-analgesic techniques. The anterior branches of spinal nerves T7-L1, running through the transversus abdominis plane, dominate the sensation of skin, muscle and parietal peritoneum [6, 17], which could be interdicted by wide-range TAP block. Subcostal TAP blocks afferent neural input and reduces central sensitization, which is consistent with the fact that local anesthetics inhibit both non-nociceptive and nociceptive stimuli. It has been reported that reported that there is a more stable level of cortisol in patients undergoing laparoscopic hysterectomy combined with TAP block than without TAP block [18]. Moreover, a case report indicated that TAP block efficiently ameliorated the stress response of a six-year-old boy who suffered from Wolf-Hirschhorn syndrome [19]. Mohamed et al. [20] reported that TAP block was effective to decrease blood glucose level in children. In this study, TAP block reduced the levels of NE, E, Cor, and glucose significantly.

Surgical trauma and perioperative pain initiate neuroendocrine response, and stimulate the release of cytokines. The pro- and anti-inflammatory cytokines are crucial for acute-phase immune response after surgery. Wind et al. [21] have showed that the concentration of circulating tumor cells is the highest after the onset of surgery. Thus, the stability of immune function is essential for patients with malignant disease. IL-6, as a pro-inflammatory cytokine, induces the synthesis of CRP and other acute phase proteins within adult stem cells. Plasma level of IL-6 is reportedly related to the severity of surgical trauma [22] and closely associated with immune status. In contrast, IL-10 is involved in immunomodulation and acts as an anti-inflammatory cytokine, which could cause systemic suppression of antitumor immunity [23]. Over the past years, the anti-inflammatory effects of local anesthetic have been extensively studied [24]. The regional anesthetic technique has been reported to reduce the cytokines and attenuate perioperative immunosuppression [25, 26]. Importantly, some retrospective studies have showed that local anesthetic had a potential benefit in patients undergoing cancer surgery [27, 28]. In our study, TAP block significantly inhibited the release of IL-6 and IL-10, whereas the levels of IL-6 and IL-10 continued increasing in the control group. Additionally, study has reported that IL-6/IL-10 is potentially related to an inflammatory poor prognosis [29]. Our results showed that the ratio (IL-6/IL-10) in control group was greater than that in TAP group during postoperative 48 h. Therefore, TAP block partially attenuated surgical trauma and maintained stable cytokine levels among patients who underwent open radical gastrectomy.

Efficient analgesia of TAP block has been well documented in human studies. Recent Meta-analyses have suggested that TAP block improves postoperative pain at both rest and movement up to 24 h postoperatively independent of the surgery types [30, 31]. In a prospective study, Niraj et al. [7] revealed that continuous TAP block could provide efficient analgesia for 8 to 72 h comparable to epidural block. In consistent with that study, our results showed that the analgesic efficacy of single-injection subcostal TAP block lasted for 12 to 16 h. Previous study reported that TAP block has more advantages for high-risk patients with cardiovascular systemic disease due to its better intraoperative hemodynamic stability [32]. Currently, the concept of the enhanced recovery after surgery has been widely addressed. Zafar et al. [33] reported that TAP block allowed an accelerated recovery before a 48 h scheduled removal of the epidural catheter. Joanne et al. [34] reported that using TAP block, a 23 h hospital stay became a realistic goal after laparoscopic colorectal resection. Similarly, our study revealed that the hospital stays significantly decreased in TAP group, which supported a trend towards decreasing hospital stay with regional blockade.

## Limitations

There were some limitations in our study. First, we only enrolled patients who underwent elective open radical gastrectomy and met the inclusion criteria, which limited the external generalizability of our results. Second, we used sufentanil for analgesia, known to affect the neuroimmunoendocrine network, which may influence our results. Third, patients in control group were not given subcostal TAP block with placebo, thus our study may be an open randomized controlled trial. Fourth, different anesthetic and antalgic medications may have complex effects on the prognosis of patient, in terms of postoperative recuperation, cancer migration, invasion, and interference with immune system, but we did not evaluate the long-term clinical effects on these individuals. Therefore, our findings are preliminary and a more comprehensive trial that assesses the long-term functional outcomes is necessary.

## Conclusions

In conclusion, subcostal TAP block could inhibit the undesirable stress response and provide efficient analgesia without increasing the adverse reactions for open radical gastrectomy. For patients with malignant tumor, the relationship between reduction in stress response and long-term clinical outcomes needs to be further studied.

## Supplementary information

**Additional file 1. Figure S1.** The flow chart of this study.

## Data Availability

The data used to support the findings of this study are available from the corresponding author upon request.

## Abbreviations

ASA: American Society of Anesthesiologists
Cor: Cortisol
E: Epinephrine
Glu: Glucose
HR: Heart rate
IL: Interleukin
NE: Norepinephrine
PACU: Post-anesthesia care unit
PCA: Patient-controlled analgesia
TAP: Transversus abdominis plane
