# Supplementary figures and images for: Transversus abdominis plane block with general anesthesia blunts the perioperative stress response in patients undergoing radical gastrectomy
Source: BMC Anesthesiol. 2019 Nov 7;19:205. doi: 10.1186/s12871-019-0861-0 (PMC6839132; doi:10.1186/s12871-019-0861-0)

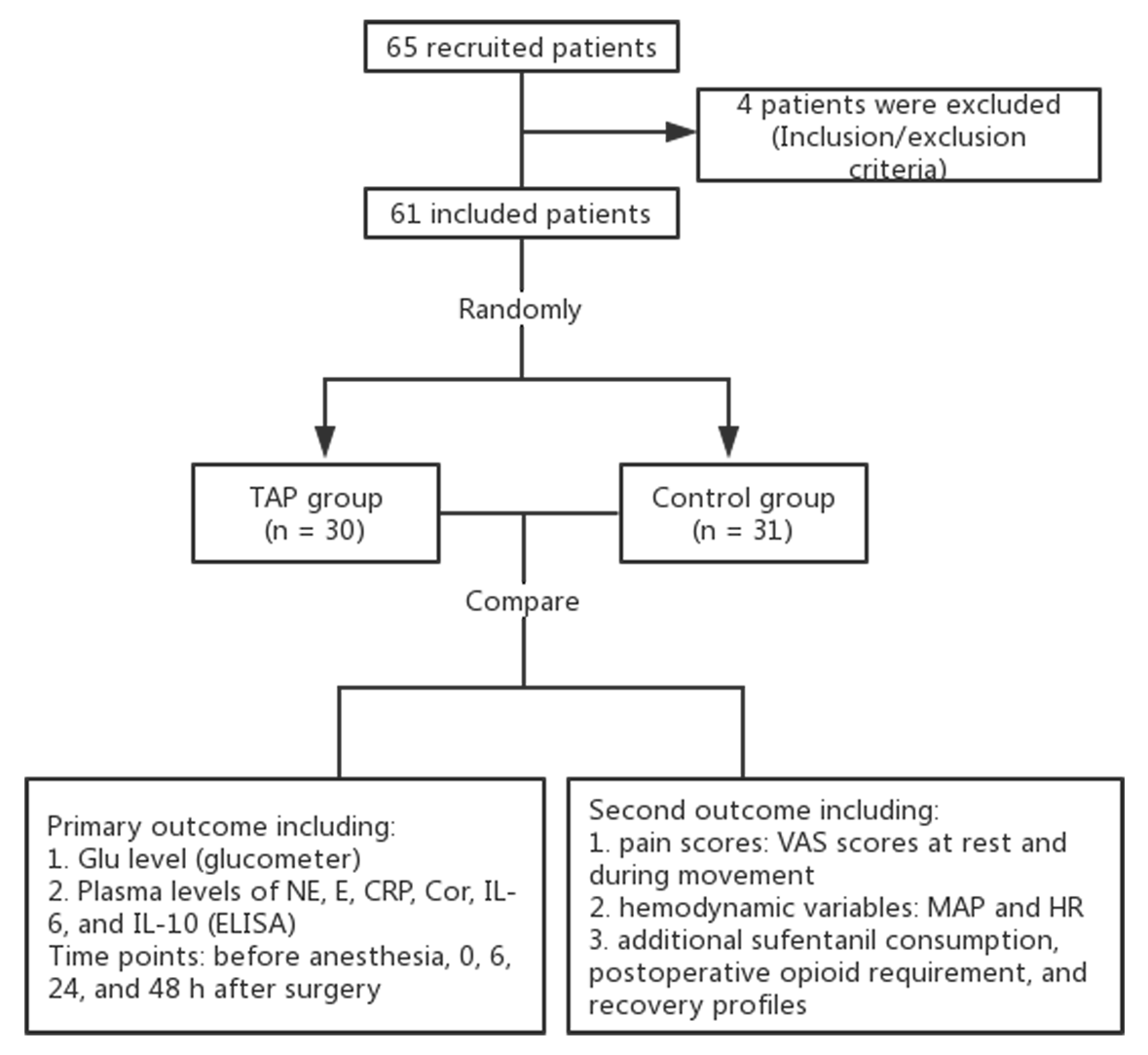

Supplement: Supplementary file 1 — Additional file 1. Figure S1. The flow chart of this study. [file 12871_2019_861_MOESM1_ESM.tif]
